# Offenders and non-offenders with schizophrenia spectrum disorders: Do they really differ in known risk factors for aggression?

**DOI:** 10.3389/fpsyt.2023.1145644

**Published:** 2023-04-17

**Authors:** Johannes Kirchebner, Steffen Lau, Lena Machetanz

**Affiliations:** Department of Forensic Psychiatry, University Hospital of Psychiatry Zurich, Zurich, Switzerland

**Keywords:** aggression, aggressive behavior, offender patient, forensic psychiatry, machine learning, model building, schizophrenia spectrum disorder

## Abstract

**Introduction:**

Individuals with schizophrenia spectrum disorders (SSD) have an elevated risk for aggressive behavior, and several factors contributing to this risk have been identified, e. g. comorbid substance use disorders. From this knowledge, it could be inferred that offender patients show a higher expression of said risk factors than non-offender patients. Yet, there is a lack of comparative studies between those two groups, and findings gathered from one of the two are not directly applicable to the other due to numerous structural differences. The aim of this study therefore was to identify key differences in offender patients and non-offender patients regarding aggressive behavior through application of supervised machine learning, and to quantify the performance of the model.

**Methods:**

For this purpose, we applied seven different (ML) algorithms on a dataset comprising 370 offender patients and a comparison group of 370 non-offender patients, both with a schizophrenia spectrum disorder.

**Results:**

With a balanced accuracy of 79.9%, an AUC of 0.87, a sensitivity of 77.3% and a specificity of 82.5%, gradient boosting emerged as best performing model and was able to correctly identify offender patients in over 4/5 the cases. Out of 69 possible predictor variables, the following emerged as the ones with the most indicative power in distinguishing between the two groups: olanzapine equivalent dose at the time of discharge from the referenced hospitalization, failures during temporary leave, being born outside of Switzerland, lack of compulsory school graduation, out- and inpatient treatment(s) prior to the referenced hospitalization, physical or neurological illness as well as medication compliance.

**Discussion:**

Interestingly, both factors related to psychopathology and to the frequency and expression of aggression itself did not yield a high indicative power in the interplay of variables, thus suggesting that while they individually contribute to aggression as a negative outcome, they are compensable through certain interventions. The findings contribute to our understanding of differences between offenders and non-offenders with SSD, showing that previously described risk factors of aggression may be counteracted through sufficient treatment and integration in the mental health care system.

## 1. Introduction

In contrast to common beliefs among experts in the 1980ies (1), there is by now robust evidence that individuals with a diagnosis of schizophrenia spectrum disorders (SSD) have a higher risk of behaving violently and aggressively than the general population: Fazel et al. e. g. reported risk estimates (OR) up to seven for men, and even up to 29 for women – although the extent of the risk elevation depends on the presence of further mediators and is rather small when influenced solely by mental disorder itself (2, 3). While there is still uncertainty about the exact mechanisms, aggression in SSD is considered to be a multifactorial phenomenon that is quite heterogenous regarding its origins (4). Potential pathways to aggressive behavior include responses to severe positive symptoms, victimization and similar adverse experiences, comorbid antisocial personality traits or disorder and/or substance use disorder, aggressive reactivity due to impaired impulse control as well as decreased ability for emotional processing (5–8). Due to their higher risk of violent behavior, individuals with SSD are also over-represented in offender populations (9).

Risk factors for aggressive and violent behavior have been the subject of extensive forensic and general psychiatric research, and several have been identified as significantly increasing its likelihood: In three large systematic / structured reviews, identified risk factors included comorbid substance use disorders and recent drug misuse, non-adherence with psychological and pharmacotherapeutic treatment, hostile behavior and poor impulse control as well as past criminal behavior (2, 10, 11). With offender patients with SSD showing a higher prevalence of aggression than non-offender patients, one might be inclined to deduce that they show a higher expression of said risk factors (12). However, comparative research exploring differences between offender and non-offenders with mental health issues is scarce, and mostly conducted in populations with mixed diagnoses and small case numbers. At the same time, findings from one group cannot be directly applied to the other, as there are several structural differences: First of all, offender patients have a higher prevalence of psychiatric comorbidities, thus complicating treatment courses (3, 13). Secondly, while aggression occurs at an increased rate in patients with SSD, forensic psychiatric patients have an additionalhistory of offending and violence and consequently involvement with the judicial system, whereas aggressive incidents committed by general psychiatric patients may be less severe and thus less likely to actually be reported to the authorities (14, 15). Lastly, offender patients are treated within a compulsory context, as the institutionalization is court-mandated and oftentimes against the will of the affected patient (14). This raises the fundamental research question whether offenders significantly differ from non-offenders with SSD in certain characteristics, and if so, in which. There is still no consensus as to whether all patients with SSD have the potential to become (violent) offenders, or whether this only applies to a subpopulation of SSD patients, in which offending is an expression of a basal structural deficiency in the sense of a “criminal heboid” (16).

To close the research gap between offenders and non-offenders, we pursued two goals with our study:

I) to identify key differences in offender patients (OP) and non-offender patients (NOP) regarding aggressive behavior through application of supervised machine learning,
II) to quantify the performance of the model.

## 2. Materials and methods

This study was reviewed and approved by the Ethics Committee Zurich, Switzerland [Kanton Zürich] (committee’s reference number: KEK-ZH-NR 2014–0480). As this study was conducted as part of a larger ongoing scientific project on fundamental research in offender patients with SSD, and as a similar methodology was applied, parts of the following section may be replicated, e. g. in (17–19).

### 2.1. Participants

We defined a forensic psychiatric study group and a general psychiatric comparison group. Our study sample comprised 370 male (91.6%) and female (8.4%) offender patients with a diagnosis of SSD (F2x acc. to ICD-10, respectively, 295.x acc. to ICD-9) who had been in inpatient treatment between 1982 and 2016 at the Center for Inpatient Forensic Therapies of the University Hospital of Psychiatry Zurich, Switzerland (20, 21). Most patients from this sample had been hospitalized from the year 2000 on (296 cases). Offenses leading to the referenced forensic psychiatric hospitalization included both violent crimes—(attempted) homicide, assault, violent offenses against sexual integrity, robbery, and arson —and/or non-violent crimes —threat and coercion, property crime without violence, criminal damage, traffic offenses, drug offenses, and illegal gun possession. Patients with non-violent offenses were included as we intended to map the entire criminal landscape encountered in forensic psychiatric patients with SSDS and, as described above, as the aim of this study was not to compare aggressive to non aggressive patients, but to conduct a comparative study on offenders and non-offenders regarding risk factors for aggressive behavior.

The comparison group consisted of 370 non-offender patients (NOP) with SSD, who had been in inpatient treatment at the Center for Integrative Psychiatry of the University Hospital of Psychiatry Zurich. We deemed this population particularly suited for comparison as, like the forensic psychiatric patients, a) they majorly comprised patients with chronic and prolonged courses of disorder, and b) they had already an established pharmacotherapy upon admission, as they were usually transferred from acute psychiatric wards – which was also true for most OP, who had been initially treated in a prison or custodial setting. The study and comparison group were matched by gender.

### 2.2. Defining the outcome variable

The outcome variable “non-offender patient (NOP)” was dichotomized as (a) true, and (b) false, with “non-offender patient –true” being defined as the positive class in further analysis.

### 2.3. Defining the predictor variables

All predictor variables included in the statistical analysis were selected in accordance with previous findings. Predictor variables included items from the following domains: sociodemographic, illness-related factors, psychopharmacotherapy, adverse events during the referenced hospitalization, childhood/youth, and physical illness. Table 1 provides an overview over our selected variables and their reference in previous literature, with some being dismissed later due to a large quantity of missing values (see 2.5). For a specific definition of the predictor variables, please refer to the coding protocol provided in the data availability statements. Items were tested for multicollinearity through calculation of the variance inflation factor (VIF). Apart from PANSS-subscales, which naturally correlated with the total PANSS-score, and items regarding country of origin, there was little to moderate correlation between the other variables.

**Table 1:** Overview over selected predictor variables and their reference in previous literature.

Variable in current study	Previous literature w. similar variable	Population: Description / sample size (*n*)
*Sociodemographic data*		
Age at admission to referenced hospitalization	(22)	NOP with SSD/ 1,410
	(23)	NOP with SSD + depression / 132
	(24)	NOP with SSD / 150
Gender	(22)	NOP with SSD / 1,410
	(23)	NOP with SSD + depression / 132
	(24)	NOP with SSD / 150
	(25)	OP with SSD / 223
Country of birth: Switzerland	–	–
Marital status^1^	(26)	NOP with SSD / 1,549
Living situation	(27)	NOP with SSD / 1,512
Level of education^1^	(26)	NOP with SSD / 1,549
	(27)	NOP with SSD / 1,512
Social network	(28)	NOP with SSD / 60
Existent low ability^1^	(29)	NOP with SSD / 253
	(27)	NOP with SSD / 1,512
	(28)	NOP with SSD / 60
*Psychiatric data*		
Age of onset of illness	(29)	NOP with SSD / 253
Comorbid alcohol use disorder	(28)	NOP with SSD /60
Comorbid substance use disorder	(28)	NOP with SSD /60
Comorbid personality disorder	(10)	n/a (systematic review)
Previous psychiatric treatment Inpatient outpatient	(29)	NOP with SSD / 253
Previous compulsory measures^1^	(27)	NOP with SSD / 1,512
Cognitive impairment	(30)	NOP and OP / 78
Delusions	(31)	NOP with SSD / 63
	(22)	NOP with SSD/ 1,410
Hallucinations	(22)	NOP with SSD/ 1,410
	(32)	NOP with SSD / 280
Penetrability of own ego	–	–
Disorders of affect/drive	(33)	Prisoners / 675
Negative symptoms	(22)	NOP with SSD/ 1,410
*PANSS: P1 -P7	(34)	OP with SSD / 352
	(26)	NOP with SSD / 1,549
	(31)	NOP with SSD / 63
^*^PANSS: N1 –N7	(34)	OP with SSD / 352
	(26)	NOP with SSD / 1,549
	(31)	NOP with SSD / 63
^*^PANSS: G1 –G16	(34)	OP with SSD / 352
	(26)	NOP with SSD / 1,549
	(31)	NOP with SSD / 63
^*^PANSS: total	(34)	OP with SSD / 352
	(26)	NOP with SSD / 1,549
	(27)	NOP with SSD / 1,512
	(31)	NOP with SSD / 63
	(22)	NOP with SSD / 1,410
Insight reg. Psychiatric disorder^1^	(31)	NOP with SSD / 63
	(28)	NOP with SSD / 60
Previous suicide attempts	(10)	n/A (syst review)
History of self-harm	(10)	n/a (syst review)
History of endangerment of others	(27)	NOP with SSD / 1,512
	(28)	NOP with SSD / 60
	(31)	NOP with SSD / 63
*Data reg. pharmacotherapy*		
Regular intake of medication as prescribed	(26)	NOP with SSD / 1,549
Non-adherence with psychotherapy	(10)	n/a (systematic review)
Olanzapine equivalent	(28)	NOP with SSD / 60
*Data reg. hospitalization*		
Negative behavior toward staff / other patients	(34)	OP with SSD / 352
Verbal/physical aggression	(35)	NOP / 254
Complains about staff	(34)	OP with SSD / 352
Dis/antisocial behavior	(34)	OP with SSD / 352
Rule-breaking	(34)	OP with SSD / 352
*Data reg. childhood / youth*		
Physical abuse during childhood^1^	(10)	n/a (systematic review)
Parental history of alcohol / drug misuse^1^	(10)	n/a (systematic review)
*Other*		
Physical / neurological illness	(26)	NOP with SSD / 1,549

* PANSS, Positive and negative syndrome scale. For this purpose, we applied an adapted three-tier scale instead of the usual seven-tier scale (36). OP, offender patients; NOP, non-offender patients; SSD, schizophrenia spectrum disorder.

1 Items later dismissed during statistical analysis due to missing values > 33%. For a detailed list of all variables included in this study as well as their definition please refer to the information in the data availability statement.

### 2.4. Data extraction

Data from the files of all patients were retrospectively assessed through directed qualitative content analysis (24). Data extraction was performed by two experienced psychiatrists according to a rating protocol based on a set of criteria originally proposed described by Seifert et al. (37). The comprehensive case files included professionally documented medical histories, psychiatric/psychologic reports inpatient and outpatient reports of both hospitalizations as well as outpatient treatments, extensive progress reports by clinicians, nursing and care staff, as well as – for the OP population – testimonies, court proceedings and data regarding previous imprisonments and detentions.

### 2.5. Data analysis

**Figure 1:**
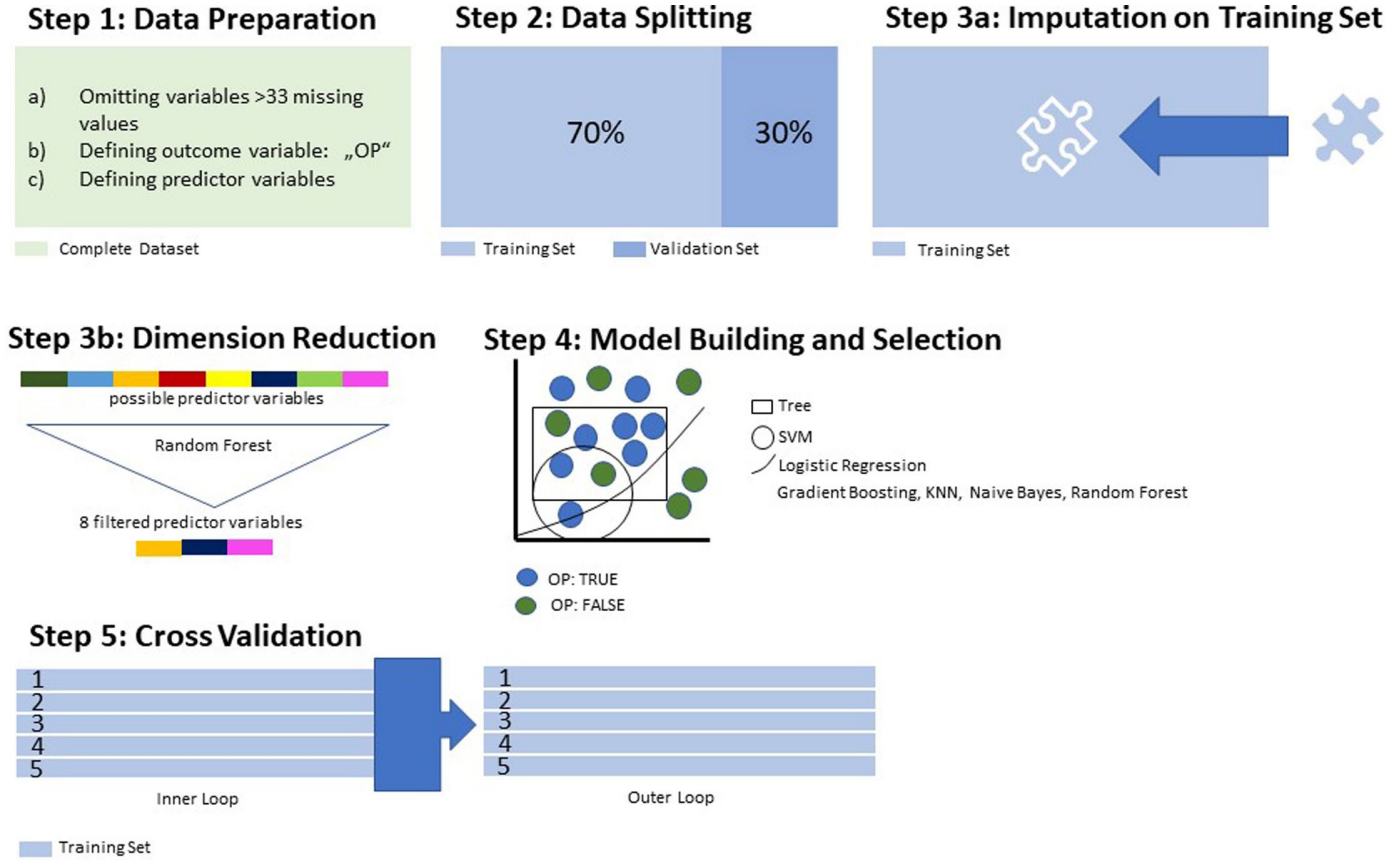
Data processing and training.

**Figure 2:**
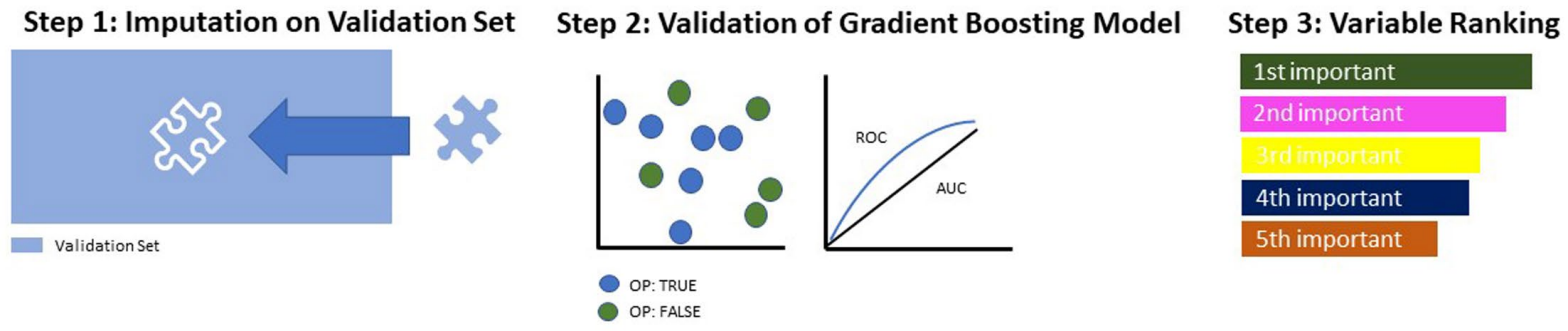
Model building and testing on validation set.

As our goal was to exploratively identify which of the possible predictor variables were most dominant in a model discriminating between OP and NOP, we applied supervised machine learning (ML) for the statistical analysis. Figures 1, 2 provide a step-by-step overview over the statistical procedures, which are further elaborated on in detail below.

Preprocessing of the data for ML included the elimination of variables with >33% of missing values, the conversion of categorial variables to binary code and the definition of the outcome variable/positive class (Figure 1, Step (1). After this data preparation, the complete dataset was split in one training subset and one validation subset. While the validation subset was stored aside, the training subset, containing 70% of all cases, was then used for the building of the model. Missing values in the predictor variables were imputated by mean and mode (Figure 1, Step (3a). To spare computational resources and increase the overall performance of the model, we reduced the number of variables through application of a random forest algorithm (Figure 1, Step (3b). This reduction in dimensionality was performed up to the point where the AUC did not improve by >5% by adding another variable. Following these preprocessing procedures, seven different algorithms were applied to the training set for discriminative model building, out of which the most suitable was selected via ROC parameters (Figure 1, Step (4). To overcome bias in performance evaluation, we performed a 5-fold cross-validation (Figure 1, Step (5). Then followed the model building and testing on the validation subset previously split from the training subset (see Figure 2).

First, just like on the training set, missing values were imputated using the same weights (Figure 2, Step (1)). Then, the most suitable model (identified in Figure 1, Step (4) was applied and evaluated in terms of its performance parameters (Figure 2, Step (2). Lastly, all identified predictor variables were ranked according to their indicative power within the model (Figure 2, Step (3)).

## 3. Results

### 3.1. Descriptive data

Both groups showed a similar age (OP: mean = 34.2 yrs., SD = 10.2; NOP: mean = 36.2 yrs., SD = 12.2). Compared to NOP, OP were more likely to have been born outside of Switzerland and to be single at the time of their admission to the referenced hospitalization. Both groups had an equal distribution of diagnoses, with paranoid schizophrenia (ICD-10: F20.0) being the most frequent, and others (hebephrenic schizophrenia, schizoaffective disorder, and other diagnoses from the schizophreniform spectrum) accounting for roughly 20% in both groups. OP were more likely to have a co-diagnosis of substance use disorders and personality disorders. They also had a higher ratio of aggressive behavior in the past as well as during the referenced hospitalization (see Table 2).

**Table 2:** Descriptive characteristics in both groups.

	Offender patients n/N (%)	Non-offender patients n/N (%)
Gender: male	339/370 (91.6)	339/370 (91.6)
Country of birth: Switzerland	167/370 (45.1)	245/367 (66.8)
Single	297/364 (81.6)	282/364 (77.5)
Diagnosis: paranoid schizophrenia	294/370 (79.5)	287/370 (77.6)
Co-diagnosis: substance use disorder	269/200 (72.9)	183/327 (56)
Co-diagnosis: personality disorder	47/370 (12.7)	26/370 (7)
History of aggressive behavior prior to referenced hospitalization	259/356 (72.8)	200/321 (62.3)
Aggressive behavior during referenced hospitalization	113/352 (32.1)	67/360 (18.6)

*n* = subgroup with characteristics; *N* = total study population.

### 3.2. Model calculation and performance measures

After application of seven algorithms on the training set, gradient boosting emerged as most suitable for the dataset with a balanced accuracy of 81.1% and an AUC of 0.91. With a sensitivity of 79% and a specificity of 83.2%, OP were correctly identified in over 4/5 the cases (see Table 3).

**Table 3:** ML models and their performance measures in nested cross-validation.

Statistical procedure	Balanced accuracy (%)	AUC	Sensitivity (%)	Specificity (%)	PPV (%)	NPV (%)
Logistic Regression	77.4	0.87	73.2	81.6	78.9	76.3
Tree	80.5	0.84	82.9	78.1	77.7	82.5
Random Forest	77.4	0.89	73.7	81.1	78.7	76.3
**Gradient Boosting**	**81.1**	**0.91**	**79**	**83.2**	**81.8**	**81**
KNN	79.8	0.85	84.2	75.5	76.3	83.5
SVM	78.2	0.88	74.8	81.6	79.6	77.6
Naive Bayes	77.8	0.88	82.3	73.3	74.5	81.3

AUC, area under the curve (level of discrimination); PPV, positive predictive value; NPV, negative predictive value; KNN = k-nearest neighbors; SVM, support vector machines. Bold font is supposed to highlight/emphasize the algorithm with the best performance measures, in this case gradient boosting.

Out of 69 possible predictor variables included in the final analysis, i.e., after omission of variables with too many missing values, 8 emerged as most predictive in a model where the AUC did not improve by >5% by adding another variable (see Table 4).

**Table 4:** Absolute and relative distribution of relevant predictor variables.

Variable code	Variable description	OP n/N (%)	NOP n/N (%)
SD3a	Country of birth: Switzerland	167/370 (45.1)	**245/367 (66.8)**
SD7a	No compulsatory school graduation	**89/342 (26)**	18/320 (5.6)
SD13	Preexisting physical or neurological illness	25/359 (7)	**135/331 (40.8)**
PH18a	Outpatient psychiatric treatment(s) before referenced hospitalization	179/340 (52.6)	**275/326 (84.4)**
PH19a	Inpatient psychiatric treatment(s) before referenced hospitalization	259/351 (26.2)	**342/362 (94.5)**
PH23p	Medication compliance (in psychiatric history)	23/204 (11.3)	**166/304 (54.6)**
DZ12	Failures during temporary leave during referenced hospitalization	64/243 (26.3)	**154/324 (47.5)**
R9e	Olanzapine equivalent at discharge in mg (mean)	**22.1 mg (SD:12.3)**	19.3 mg (SD: 14.2)

*n*, subgroup with characteristics; *N*, total study population; SD, Standard deviation; PANSS, positive and negative syndrome scale; OP, offender patients; NOP, non-offender patients. Bold font indicates dominance a higher expression of the item in comparison to the other group. For a detailed definition of each of these variables, please refer to appendix. Bold font indicates higher expression of the specific variable in the group.

### 3.3. Final model performance

When applied to the validation subset (30% of all cases), the gradient boosting algorithm yielded a balanced accuracy of 79.9% and an AUC of 0.87. With a specificity of 82.5%, which was nearly the same as on the training subset, OP were again correctly identified in slightly over 4/5 the cases (see Table 5). As the validation set did not undergo the same data processing steps as the test set, and therefore did not provide optimal conditions for the algorithm, the performance parameters were a bit lower, but still meaningful.

**Table 5:** Performance measures of final gradient boosting model on validation set.

Performance measures	% (95% CI)
Balanced accuracy	79.9 (73–84.4)
AUC	0.87 (0.82–0.92)
Sensitivity	77.3 (68.5–84.3)
Specificity	82.5 (73.5–89)
PPV	83.6 (75.1–89.8)
NPV	75.9 (66.7–83.3)

AUC, area under the curve (level of discrimination); PPV, positive predictive value; NPV, negative predictive value.

### 3.4. Ranking of predictor variables

**Figure 3:**
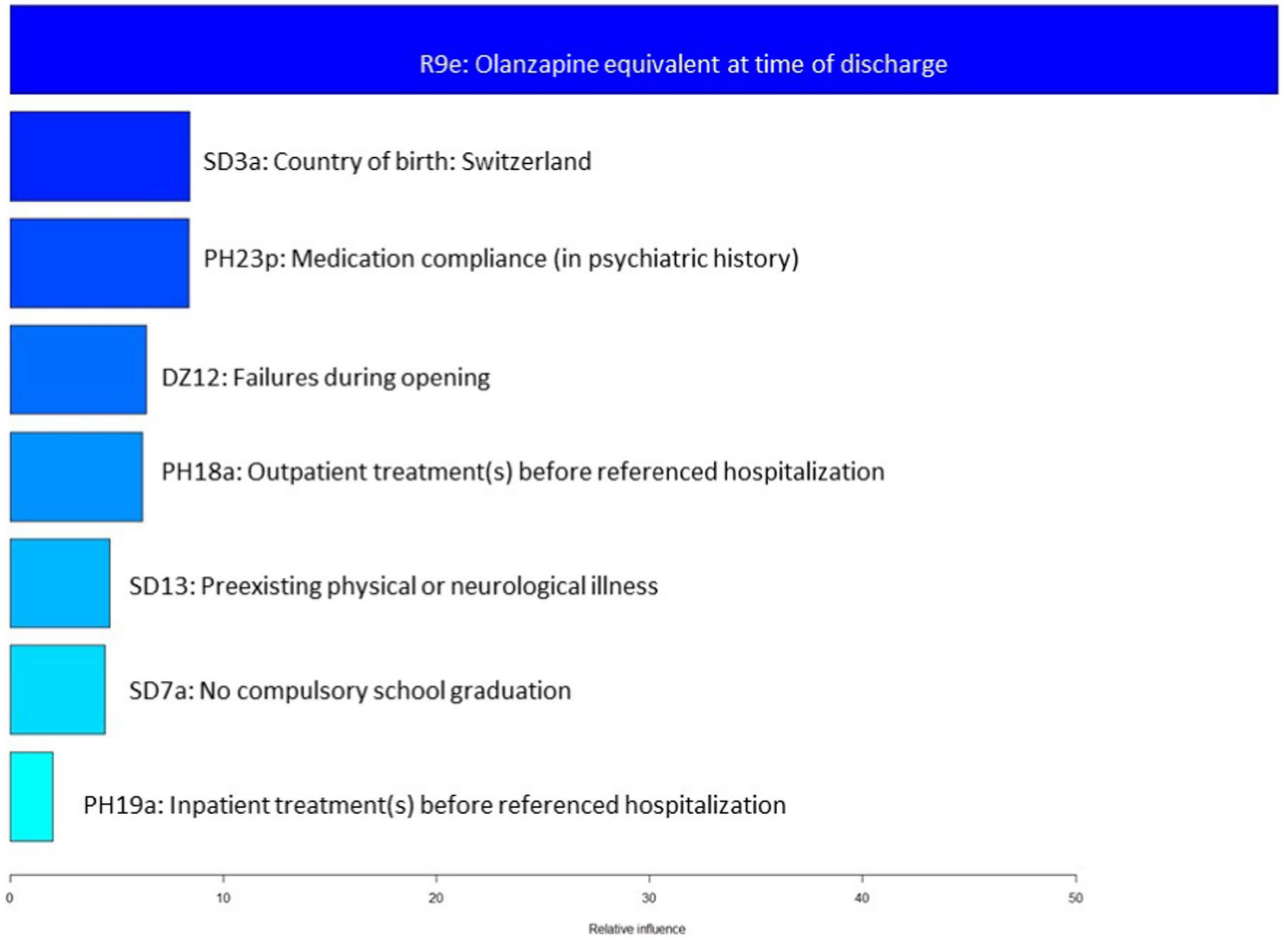
Variable importance ranked by gradient boosting.

When ranked in accordance with their indicative power within the model, the olanzapine equivalent upon discharge proved to be most dominant, with the other variable having a similar weight (see one-sided tornado graph, Figure 3).

## 4. Discussion

While certain risk factors of aggressive and criminal behavior among patients with SSD are well researched, research into the question of which individuals come into conflict with the law is still in its infancy. The goal of our study was therefore to explore risk factors of aggression in a sample of both offender and non-offender patients with SSD and evaluate which of these factors are the most distinguishing between the two groups. As expected, OP showed a higher rate of aggressive behavior both before and during the current hospitalization. Since we dealt with a large quantity of possible predictors (69 items), we deemed supervised machine learning (ML) most suitable as statistical approach, with gradient boosting yielding the best performance parameters: With a balanced accuracy of 79.9%, an AUC of 0.87, a sensitivity of 77.3% and a specificity of 82.5%, the model was able to correctly identify offender patients among the total study population in over 4/5 the cases.

The olanzapine equivalent upon discharge from the referenced psychiatric hospitalization emerged as variable with by far the highest indicative power in our model: With a mean cumulative dose of 22 mg, OP were subjected to higher doses of antipsychotic substances than NOP. The finding of higher antipsychotic doses is in line with previous literature: In a comparative study of violent and non-violent inpatients with SSD, Ellouze et al. found the former to be characterized by significantly higher neuroleptic doses (28). From a clinical perspective, it seems logical at first glance that patients with a tendency of aggressive behavior receive higher antipsychotic doses. However, as pointed out in a previous publication by our research group using the same sample, OP and NOP did not significantly differ in psychopathology, leaving the question for the rationale behind higher antipsychotic prescriptions unanswered (19). This suggests that factors unrelated to psychopathology may influence prescription decisions and lead to higher antipsychotic doses in offender populations (38). For instance, it could be hypothesized that due to forensic-psychiatric patients’ history of -sometimes severe –violence, clinicians are less reluctant to prescribe antipsychotics even in higher doses with the goal of avoiding aggressive behavior in a population already at high risk for violence. Another explanation may lie in the dual mandate of the forensic psychiatrist: In contrast to general psychiatry, the treatment of offenders in the context of court-mandated therapy is not only about the individual needs of the patient, but also about the safeguarding of society, i.e., the prevention of further crimes by the person concerned (39, 40). Assuming that the mental disorder conditioned the crime committed, this aspect might lead clinicians in the forensic psychiatric sector to make a higher claim for remission than might be made in a general psychiatric context. However, these possible explanations are merely hypotheses: So far, there is a lack of research on decision-making in pharmacotherapy of offender patients with SSD due to high ethical and legal hurdles for clinical research on this population that is particularly vulnerable due to their severe mental illness and the coercive treatment context.

The remaining seven variables showed a similar indicative power: While roughly 2/3 of NOP were born in Switzerland, this applied to less than half OP. This cannot be merely explained by the higher prevalence of SSD in patients with migrational background, as the diagnostic distribution among the two groups was quite similar (41, 42). Yet, it is not too surprising that migration background was among the most powerful distinguishing variables: A higher risk ratio for receiving mental hospital orders for migrants compared to the general population has been described before, and it has been hypothesized that there may be a specific bias in court rulings (43). However, another explanation seems more plausible: Non-European migrants have a higher likelihood of having had experiences that correlate with criminal behavior, such as childhood poverty or low socioeconomic status (44–47). Secondly, depending on their country of origin, migrants may encounter more difficulties in receiving mental health care due to cultural and/or language barriers (48, 49). Here, a link to other variables found to be of high indicative power in our model distinguishing OP from NOP emerges: the much lower ratio of previous psychiatric in-and outpatient treatments and of pharmacotherapeutic compliance in the OP group. Insufficient integration into the therapeutic network of helpers and mental health care system is known to correlate with higher rates of both violent and non-violent offending, as affected individuals are less aware of their diagnosis and may consecutively show less insight into their need for treatment and the potential risk to themselves and third parties that they may pose (19, 50, 51). As a lack of adherence to pharmacotherapy is not only associated with an increased risk of violence, but also significantly elevates the risk of relapse as well as chronification, interventions to improve insight into the need of treatment are essential (10, 52). This also applies to therapeutic components other than pharmacotherapy, such as regular consultations with psychiatric services in outpatient settings, which can serve as professional corrective in case of incipient decompensation and provide intensified support when needed, e. g. through referral to a psychiatric hospital. Patients who regularly visit a psychiatrist or psychologist may have also developed a basis of trust and thus be more likely to openly report reoccurring symptoms. Individuals less-embedded in the mental health care system as well as their relatives may in contrastnot know where to turn to in case of psychiatric deterioration and consecutive aggressive behavior, while patients with a previous history of psychiatric treatment may have developed skills for coping with crises and know to alert appropriate professional third parties (19).

Another variable quite dominant in the model was failures during temporary leave in the sense of a lack of obedience to any agreed rules while having a permission to temporarily leave an inpatient ward, e. g. through staying off the ward for longer than allowed or through consuming drugs during the leave. Failures during temporary leave were much more frequent in the NOP population. It has to be noted this finding stems most likely from structural differences in treatment: The possibilities to move freely outside the institution are much more restrictive for patients in closed forensic psychiatric institutions than for general psychiatric patients, some of whom are also in therapy on a voluntary basis and are less limited in their freedom. In addition, noncompliance with a set of rules during absences from the ward is potentially accompanied by more serious consequences in forensic psychiatric therapy up to the prolongation of treatment and thus deprivation of liberty (17). For example, the consumption of alcohol or drugs may violate a court ordered abstinence, or an attempt to abscond can lead to an extended restriction of autonomy such as the transfer to a ward with higher security measures (53). This may pose a higher threshold for OP to break rules during temporary leaves from wards. It is therefore unsurprising, that this variable proved to have a high indicative power for distinguishing between OP and NOP.

NOP were also much more likely to have a previous physical or neurological illness in addition to their psychiatric disorder. This contradicts the generally sparse findings available so far: In a Chinese sample of patients with SSD, Wu et al. found comorbid physical disease to be associated with aggressive behavior, especially cardiovascular diseases, and hypothesized that this increased risk stemmed from stress and anxiety induced by somatic problems (26). However, this particular factor could be interlinked with the higher rate of previous psychiatric treatments so far: While there is a high prevalence of (chronic) physical-health comorbidities, NOP may be more likely to consult a general practitioner or other health care professionals just as they were more likely to be in psychiatric treatment, as opposed to OP who may have a higher reluctancy to engage with the health care system. In turn, physical health issues may be under-recognized in OP populations if they are less willing to approach medical aid or to undergo diagnostic procedures, meaning that our OP population may be subjected to a significant number of unreported/unidentified case (54).

Finally, ranking second to last in our model, the variable “no compulsory school graduation” showed a higher expression in the OP population. In previously published literature, there too is some evidence for a correlation between violent and non-violent offending and low academic achievement or educational attainment, although it in the population of patients with SSD, associations seem to be weaker (47, 55).

Interestingly, variables directly linked to the frequency or severity of violent behavior did not emerge as highly dominant within the model: When considering the descriptive characteristics, OP had a much higher expression of both aggressive behavior in the past as well as during the referenced hospitalization. Yet, neither of these items had a high indicative power in distinguishing between OP and NOP. This is not self-explanatory, especially as we included not only violent offenders but also non-violent offenders in our analyses. While a history of violent criminal behavior and a higher expression of both verbal and physical aggression has been demonstrated to significantly increase the risk of violent behavior in psychotic disorders, it does not seem to divide offender from non-offender patients (10). Another striking finding was that none of the items emerging as dominating and defining the model differentiating between the two groups referred to psychopathology –e. g. the presence of positive or negative symptoms or cognitive impairment. This seems to be counterintuitive when looking at previous findings: For instance, Swanson et al. described a correlation of positive psychotic symptoms and violence, as did other authors (22, 26, 31, 32, 56). Ahmed et al. found cognitive deficits to increase the risk of aggressive behavior in patients with schizophrenia (30). Furthermore, comorbidities, such as substance use and personality disorders, both well established as mediators of aggressive behavior in SSD patients, are known to be more prevalent in offender populations than in general psychiatric samples, and have also been found to increase the likelihood of involvement with the judicial system (6, 57–59). Therefore, one would think that these items would weigh strongly in a model differentiating between OP and NOP –so why do our findings point in a different direction?

When looking for an explanation, it seems noteworthy to stress that –as one of its biggest statistical strengths –machine learning allows the evaluation of the mutual interplay of various variables with each other. When variables are no longer evaluated as stand-alone but in the context of a large set of items, they may be influenced by other variables in the calculation and therefore receive a different evaluation in the overall view of all variables included in the analysis. Bearing this in mind, our findings suggest that while comorbidities and certain psychopathologic traits as stand-alone factors increase the likelihood of aggressive behavior, they may not necessarily increase the likelihood of consecutive involvement in the judicial system and seem to be compensable through other factors when considered as whole model, e. g. sufficient embedding in the mental health care system. As described above, a history of both in-and outpatient treatments prior to the referenced hospitalization had a high indicative power in distinguishing NOP from OP, thus suggesting that established treatment structures have a preventive character regarding offending and involvement with the judicial and penitentiary system. The same can be inferred for pharmacotherapeutic compliance, which as outlined above also turned out to be dominant within the model. This interpretation leads to direct implications for clinicians in the sense that it stresses the importance of early detection of mental illness, the according integration into appropriate health care structures as well as the importance of promoting awareness of mental disorder, individual risk factors and need for treatment in those affected.

Our findings are also significant to health policy: Patients with SSD, especially those with additional vulnerability factors and risk factors for aggressive behavior, should have extremely low hurdles when accessing mental health services and support structures (e. g. bureaucratically, financially). This applies even more so to disadvantaged population, such as migrant patients or patients from low socioeconomic backgrounds.

### 4.1. Strengths and limitations

A major strength of this study lies within the direct comparison of a rather homogenous sample of 370 offender patients with 370 non-offender patients with SSD. To date, there appears to be no other comparative study on correlates of aggression in such a specific yet forensic-psychiatrically relevant population. Another advantage of our methodology lies in the application of ML: As opposed to most commonly used statistical approaches, ML allows the analyses of a large quantity of variables as well as their interplay in a multidimensional model, making it ideal for exploring phenomena that are not monocausal, but influenced by various dimensions in a multifactorial way (60, 61). As described above, individual factors may increase the risk of aggressive behavior on their own but lose some of their significance in combination with other influencing factors.

However, one has to be aware of the limitation of our study, most obvious, the retrospective design, which is inferior to prospective analyses regarding risks of bias, e. g. from resources. The reduction of sometimes complex variables to a dichotomized form may have also led to loss of information to some extent. This applies specifically to variables that lack clear definitions, such as “history of aggression,” which, e. g. in their severity, may be interpreted differently when documented by different clinicians in the files of our population. Also, as it is often the case with retrospective studies, some variables of interest in the context of aggressive behavior had a high number of missing values, and thus had to be excluded from our analysis. This applied for instance to childhood variables such as parental neglect or abuse, both of which have been described to at least moderately increase the risk of aggressive behavior in patients with SSD and would have been interesting to investigate regarding their influence in our model (10). Furthermore, machine learning algorithms perform best on large datasets. While our population of 740 patients can be considered large from a forensic-psychiatric point of view, it is a rather modest quantity for ML purposes. Thus, and although we employed a nested cross-validation, the model is more likely to be subjected to overfitting than it would have been in a larger sample (62). Another statistically relevant limitation that should be noted is the multicollinearity between all items regarding the PANSS score as well as items regarding the patients’ country of origin, which may have created redundant information in the model and led to skewed results and thus a decrease of the power of the model. Also, it should be noted that while the higher mean dose of antipsychotics in the OP group was not explicable through a more severe degree of psychopathology, it may have resulted from the elevated expression of aggressive behavior during and prior to the hospitalization. Thus, aggression may have indirectly influenced the model without showing up as one of the most influential predictor variables.

Lastly, one has to be cautious to directly derive causality from our findings before the identified model is applied to other populations for validation. As it is the case with offender populations, with only 8.4%, our sample lacked a number of female patients large enough to derive implications specifically for women with SSD. Finally, our offender sample consisted of both non-violent and violent offenders, and different indicative factors may emerge when only the latter are analyzed. However, we decided not to exclude female patients and non-violent offenders in order to represent a patient population that most closely corresponds to the reality of the mass penal system.

### 4.2. Conclusion

In summary, our study was able to contribute to a better understanding of differences between offender and non-offender patients with SSD: When evaluating factors linked to aggressive behavior, antipsychotic dose, integration in and compliance with the mental health system, migrant background, low level of education and physical/neurological illness emerged as most indicative, while items related to psychopathology and aggression itself did not show a heavy influence on the model. While static variables such as migrant background cannot be therapeutically influenced directly, these findings suggest that other known risk factors, such as comorbid substance use disorders, are compensable through sufficient psychiatric treatment. Furthermore, our findings challenge the previous knowledge on risk factors of aggressive behavior: Even though for instance comorbid substance use disorders, positive symptoms or a history of violent behavior are well established as increasing the risk of aggression in patients with SSD, they are not dominant dividers between offenders and non-offenders. This raises the question of whether these factors should really be given such great significance in risk assessment, or whether, in the interplay of various influencing factors, more attention should be paid to other domains.

However, as this is merely an explorative analysis on a particular subpopulation, our results do not allow direct causal inferences. Therefore, the authors recommend validation of these findings in further, preferably larger populations. If proven robust, our results advocate for an intensified efforts by both clinical as well as health political agents to integrate individuals suffering from SSD in the mental health care system. This applies especially to patients with a particularly high risk for violent behavior and high hurdles in reaching and approaching professional help systems. The distinct dominance of the variable “olanzapine equivalent” within the model, which seems to not be explicable by a higher severity of psychopathology in the offender sample, highlights the need for further research on prescription practices and clinical decision making in the pharmacotherapy of offenders with SSD.

## Data availability statement

The raw data supporting the conclusions of this article will be made available by the authors, without undue reservation.

## Ethics statement

The studies involving human participants were reviewed and approved by Ethics Committee Zurich, Switzerland [Kanton Zürich] (committee’s reference number: KEK-ZH-NR 2014–0480). Written informed consent for participation was not required for this study in accordance with the national legislation and the institutional requirements.

## Author contributions

JK: conceptualization, software, validation, and project administration. JK and LM: methodology, formal analysis, investigation, and data curation. JK and SL: resources. LM: writing—original draft preparation and visualization. LM, SL, and JK: writing—review and editing. SL and JK: supervision. All authors contributed to the article and approved the submitted version.

## Conflict of interest

The authors declare that the research was conducted in the absence of any commercial or financial relationships that could be construed as a potential conflict of interest.

## Publisher’s note

All claims expressed in this article are solely those of the authors and do not necessarily represent those of their affiliated organizations, or those of the publisher, the editors and the reviewers. Any product that may be evaluated in this article, or claim that may be made by its manufacturer, is not guaranteed or endorsed by the publisher.

## Appendix

**Table:**

Variable code	Variable description	Definition
SD3a	Country of birth: Switzerland	Country of birth as indicated in the file
SD7a	No compulsatory school graduation	Yes, if he/she had not completed primary or (lower) secondary school education (school period from about age 6 to about age 16) at the time of the referenced hospitalization
SD13	Preexisting physical or neurological illness	Yes, if, at the time of the investigated offense, he/she suffered from any type of pre-existing disease or injury (except for psychiatric disorders)
PH18a	Outpatient psychiatric treatment(s) before referenced hospitalization	Yes, if he/she had visited a mental health care provider (psychologist and/or psychiatrist) as an outpatient at any time before the referenced hospitalization, regardless of the duration of said treatment
PH19a	Inpatient psychiatric treatment(s) before referenced hospitalization	Yes, if he/she had visited a mental health care provider (psychologist and/or psychiatrist) as an inpatient at any time before the referenced hospitalization, regardless of the duration of said treatment
PH23p	Medication compliance (in psychiatric history)	Yes, if he/she, mental health professionals and trusted private persons (e.g., close family members) had not reported/documented a lack of compliance/adherence to any antipsychotic medications at any time before the investigated offense and if mental health professionals and trusted private persons (e.g., close family members) had not had reasonable grounds for suspecting that the patient lacked medication compliance/adherence to any antipsychotic medications at any time before the referenced hospitalization
DZ12	Failures during temporary leave	refers to a lack of obedience to any agreed rules while having a permission to temporarily leave an inpatient ward or during the opening of an (otherwise closed) inpatient ward, which includes any of the following … (a) staying away from the ward for longer than allowed (including no return at all) (b) the prohibited consumption of substances (including alcohol and prescription medications) outside of the ward (c) any other violation of agreed rules while having a permission to leave an inpatient ward or during the of an (otherwise closed) inpatient ward
R9e	Olanzapineequivalent at discharge	Cumulative dose of antipsychotic medication included in his/her prescription schedule at the time of discharge from the referenced forensic hospital converted to olanzapine equivalents in milligrams
